# A Proline-Based 'Redox–Release' Platform for Programmed Access to Diverse Sulfur(VI) and Fluoroalkyl Radicals

**DOI:** 10.21203/rs.3.rs-11001470/v1

**Published:** 2026-09-17

**Authors:** Elijah T. Marris, Amir D. Johnson-Sammy, Brandon D. Davis, Davis J. Fowler, Emanuele Casali, Dilhumar Uyghur, Nicholas P. R. Onuska, Jon I. Day, Xiaoyong Li, Giuseppe Zanoni, Jennifer M. Schomaker

**Affiliations:** 1 Department of Chemistry, University of Wisconsin-Madison; Madison, Wisconsin 53706, USA; 2 Department of Chemistry, University of Pavia, Via Torquato Taramelli, 12, 27100 Pavia PV, Italy; 3 Lilly Research Laboratories, Eli Lilly and Company, Indianapolis, Indiana 46285, United States

## Abstract

Radical identity is traditionally coupled to the strategy required for its generation, necessitating distinct precursor classes and activation modes to furnish different reactive intermediates. Here, we introduce a modular *redox-release* platform that provides programmable access to diverse S(VI) and fluoroalkyl radicals from a common proline-derived scaffold, decoupling the radical identity from precursor activation. Complementary oxidative and reductive activation pathways converge on a shared α-amino radical intermediate to furnish chemically distinct radicals. The platform exhibits broad utility in hydrofunctionalization, late-stage functionalization, synthetic diversification, and target-oriented synthesis, while mechanistic and computational studies suggest a common β-scission pathway underlying its generality. More broadly, redox-release establishes a strategy in which activation mode and radical identity become independent design parameters for radical generation.

## Introduction

**Fig. 1.**
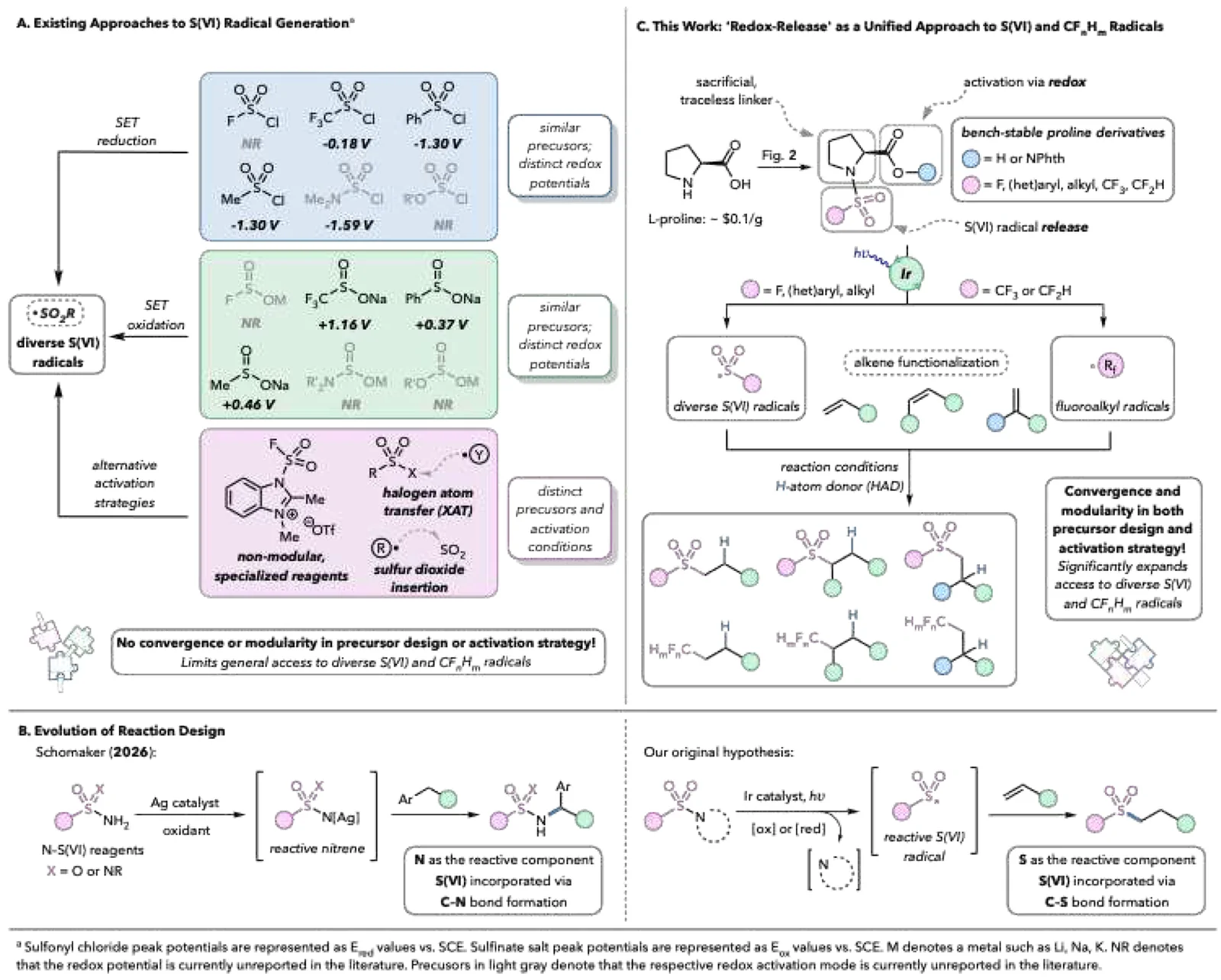
(**A**) Existing approaches to S(VI) radical generation and redox potentials of common S(VI) radical precursors. (**B**) Inspiration for this reaction design. (**C**) Development of a unified ‘redox-release’ platform for generating diverse S(VI) and fluoroalkyl radicals.

Radicals enable bond constructions that are inaccessible through conventional polar and ionic pathways, making them powerful intermediates in organic synthesis.^1–6^ However, access to radicals is frequently constrained by specific precursor structures. Even closely related radical precursors can display markedly different redox properties, thereby requiring distinct activation strategies. Others cannot be engaged through single-electron activation, instead requiring orthogonal activation modes such as halogen-atom transfer (XAT).^7–9^ As a result, radical identity is often inseparable from the strategy required to generate it, limiting efforts to develop general platforms for accessing broad classes of reactive intermediates. Sulfur(VI)-centered radicals provide a particularly instructive case. Sulfonyl, fluorosulfonyl, sulfamoyl, and other S(VI) moieties are prevalent in medicinal chemistry, chemical biology, and materials science;^10–13^ however, S(VI) radical precursors span a wide range of redox potentials owing to substituent-dependent σ-inductive and π-resonance effects at sulfur (Fig. 1A).^14,15^ Consequently, their generation requires precursor- specific activation modes that employ different catalysts and reaction conditions. Thus, a unified strategy to generate structurally diverse S(VI) radicals under conditions independent of radical identity would significantly expand the synthetic utility of these valuable motifs.

An unexpected clue to such a strategy emerged from recent advances in nitrene transfer, where structurally diverse N-S(VI) reagents converge on a shared activation pathway despite substantial variation in S(VI) substitution (Fig. 1B).^16,17^ We wondered whether an analogous principle could be applied to radical generation; specifically, could a common reagent architecture release S(VI) radicals through a shared redox activation pathway largely independent of the appended group? Herein, we report a proline-derived radical precursor platform (Fig. 1C), which we term “redoxrelease.” Redox-release is a strategy for the design and application of radical precursors in which radical identity is programmed within a common molecular scaffold and selectively released through complementary oxidative or reductive activation pathways. The platform provides systematic access to not only multiple classes of S(VI)-centered radicals, but also fluoroalkyl radicals through downstream fragmentation pathways. These studies establish N-S(VI) reagents as programmable radical precursors and demonstrate that radical generation can be decoupled from precursor-specific activation requirements, providing a general framework for releasing diverse reactive intermediates from a common reagent architecture.

## Results and Discussion

**Fig. 2.**
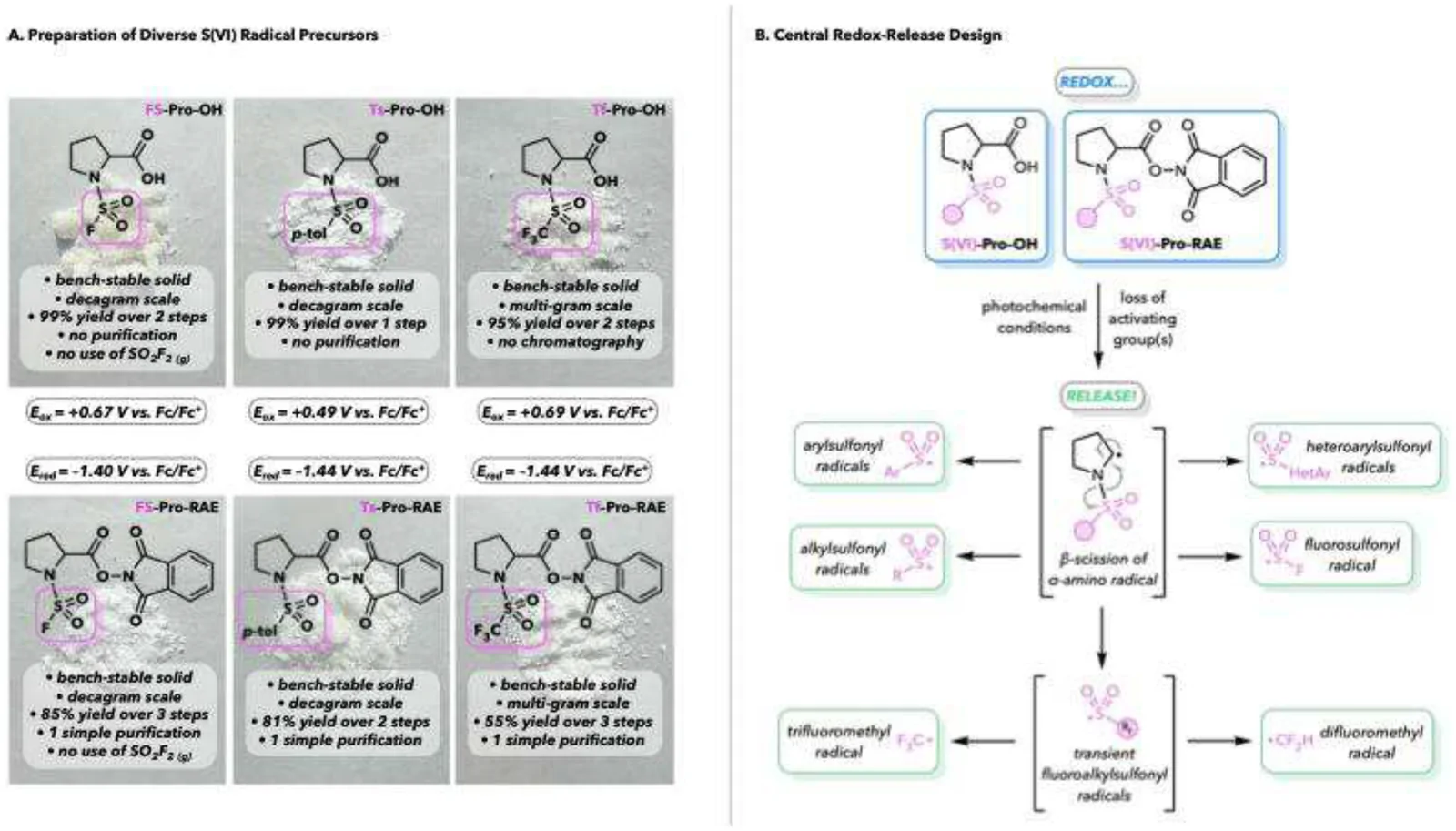
Design and preparation of reagents for the redox-release platform. (**A**) Select redox-release reagent library and corresponding onset redox potentials. Carboxylic acid redox potentials were measured in the presence of 1.0 equiv. TMG to obtain the carboxylates in-situ. (**B**) General design concept.

To implement our strategy, we designed a family of proline-derived N–S(VI) reagents that converge on a common α-amino radical intermediate following either oxidative or reductive activation (Fig. 2). The design was inspired by reports that α-amino acid sulfonamides undergo decarboxylation and N–S bond cleavage after silver-catalyzed oxidation to generate sulfonyl radicals.^18,19^ We reasoned that this fragmentation could be generalized into a modular photoredox strategy for programmable S(VI) radical release. By positioning the redox-active moiety on the amino acid backbone, the N-bound S(VI) substituent is electronically insulated from the activation site, allowing radical generation to proceed largely independently of the appended group’s intrinsic redox properties.

To evaluate the redox-release strategy, we examined the electrochemical properties of representative reagents bearing chemically distinct S(VI) substituents (see S61 in Supporting Information). In contrast to conventional S(VI) radical precursors, which span broad, precursor-dependent electrochemical windows (Fig. 1A, *vide supra*), the selected redox-release reagents showed remarkably consistent activation potentials (Fig. 2A). Cyclic voltammetry revealed that both the proline-derived redox-active esters (RAEs) and the corresponding carboxylic acids exhibit uniform reduction and oxidation onset potentials, respectively, spanning only 0.04 V (*E*_red_ ≈ −1.44 to −1.40 V vs. Fc/Fc^+^) and 0.20 V (*E*_ox_ ≈ +0.49 to +0.69 V vs. Fc/Fc^+^) across sulfonyl fluoride, aryl sulfonyl, and trifluoromethyl sulfonyl derivatives. These data support the key design premise that electrochemical activation is governed primarily by the common proline-derived redox handle, rather than the identity of the transferable S(VI) group.

A representative library of bench-stable proline-derived S(VI) radical precursors was prepared from inexpensive, commercially available starting materials (Fig. 2A). Most carboxylic acid precursors were obtained directly from L-proline through a single N-functionalization step, while the sulfonyl fluoride and trifluoromethyl sulfonyl derivatives were synthesized in concise two-step sequences from a common intermediate. The corresponding redox-active esters were then prepared in a single derivatization step in uniformly high yields. Notably, the sulfonyl fluoride precursors were accessed through a gas-free route from commercially available SuFEx-IT, avoiding sulfuryl fluoride gas and the specialized precursor syntheses required for previous fluorosulfonyl radical reagents.^20–22^ These studies established practical, modular routes to a common family of redox-release reagents for demonstrating the platform across representative radical transformations.

We anticipated that both oxidative decarboxylation of **S(VI)-Pro-OH** and reductive activation of **S(VI)-Pro-RAE** would converge on a common α-amino radical intermediate (Fig. 2B). Subsequent β-scission would convert the proline scaffold into a benign imine and release the S(VI) radical through N–S bond cleavage. Because radical generation proceeds through a shared fragmentation pathway, the inherent redox properties of the transferable S(VI) group no longer dictate the activation strategy required for release. Accordingly, the platform should provide unified access to structurally diverse S(VI)-centered radicals, including •SO_2_F, •SO_2_Ar, •SO_2_HetAr, •SO_2_R, •SO_2_CF_3_, and •SO_2_CF_2_H. Furthermore, if •SO_2_CF_3_ and •SO_2_CF_2_H undergo SO_2_ extrusion, this strategy could provide access to fluoroalkyl radicals, including •CF_3_ and •CF_2_H.

## Reaction Optimization

Alkene hydrofluorosulfonylation was selected as an initial benchmark transformation. Beyond serving as a rigorous test of fluorosulfonyl radical generation, this reaction provides efficient access to alkyl sulfonyl fluorides, an increasingly important class of S(VI)-containing compounds in medicinal chemistry. Alkyl S(VI) derivatives comprise approximately two-thirds of the S(VI) motifs found in FDA-approved small molecule drugs approved since 2011, reflecting favorable physicochemical properties that include enhanced metabolic stability, aqueous solubility, and bioactivity.^23,24^ Alkyl sulfonyl fluorides are particularly valuable because the S–F bond combines excellent stability with utility as versatile sulfur(VI)-fluoride exchange (SuFEx) handles for modular diversification into sulfonamides, sulfonates, sulfones, and other S(VI) motifs. These scaffolds also serve as privileged covalent warheads for the selective modification of nucleophilic amino acid residues in proteins.^25–27^

**Fig. 3.**
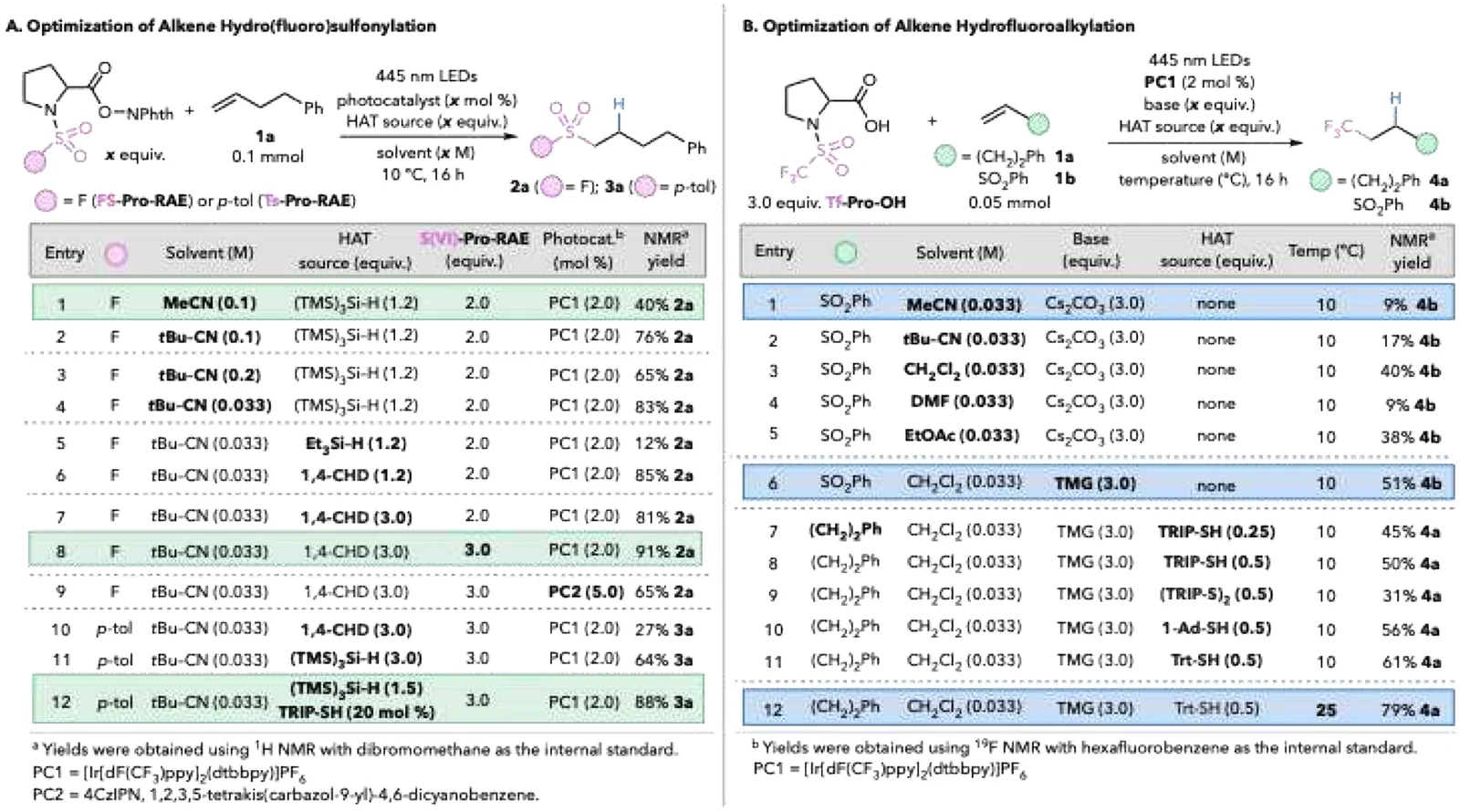
Selected reaction optimization for alkene hydrofunctionalizations. (**A**) Hydro(fluoro)-sulfonylation. (**B**) Hydrofluoroalkylation.

Using 4-phenyl-1-butene **1a** as the model substrate, single-electron reduction of **FS-Pro-RAE** in the presence of [Ir[dF(CF_3_)ppy]_2_(dtbbpy)]PF_6_ (**PC1**) and tris(trimethylsilyl)silane ((TMS)_3_SiH) furnished the desired alkyl sulfonyl fluoride **2a** in 40% NMR yield (Fig. 3A, entry 1). Systematic evaluation of the photocatalyst, hydrogen-atom donor (HAD), stoichiometry, and concentration afforded optimized conditions in entry 8, delivering **2a** in 91% NMR yield. Both 1,4-cyclohexadiene (1,4-CHD) and (TMS)_3_SiH proved competent as HADs, consistent with direct trapping of the carbon-centered radical adduct after addition of •SO_2_F to the alkene. Control experiments confirmed that light, photocatalyst, and HAT source were all required for productive reactivity (see Fig. S3 in the Supporting Information). The 4CzIPN (**PC2**) organic dye provides a cost-effective alternative to transition metal photocatalysts (entry 9).

We examined whether the same reductive activation manifold could be extended to a structurally and electronically distinct S(VI) radical. Indeed, single-electron reduction of **Ts-Pro-RAE** proceeded under the same conditions to furnish the desired product **3a** in 27% yield (Fig. 3A, entry 10). However, addition of Ts• to **1a** generates a less electrophilic carbon-centered radical than •SO_2_F, resulting in a polarity mismatch during HAT. Polarity-reversal catalysis (PRC) (Fig. 6Ac, *vide infra*) using catalytic 2,4,6-triisopropylbenzenethiol (TRIP-SH) and stoichiometric (TMS)_3_SiH provided a solution,^28^ furnishing **3a** in 88% NMR yield (entry 12). Together, these studies demonstrate that proline-derived RAEs provide a general reductive activation method for generating S(VI) radicals with distinct redox properties.

We next sought to demonstrate a complementary oxidative activation pathway using the redox-release platform. Extending this strategy beyond S(VI)-centered radicals to fluoroalkyl radicals would substantially broaden its synthetic utility given the widespread importance of trifluoromethyl groups in pharmaceuticals, agrochemicals, and functional materials.^29,30^ Although either the acid or RAE precursor can participate in redox-release (see Fig. S4 in the Supporting Information), **Tf-Pro-OH** was selected for initial optimization with phenyl vinyl sulfone **1b** as a representative Giese acceptor. While oxidative decarboxylation of **Tf-Pro-OH** was expected to generate the corresponding α-amino radical, it was not clear whether the subsequent double β-scission events to release •CF_3_ would prove successful. Encouragingly, hydrotrifluoromethylation of **1b** resulted in **4b**, albeit in a low 9% yield (Fig. 3B, entry 1). Optimization identified Cs_2_CO_3_ (entry 3) and tetramethylguanidine (TMG) (entry 4) as effective bases, with dichloromethane as the optimal solvent, ultimately increasing the yield of **4b** to 51%.

**Fig. 4.**
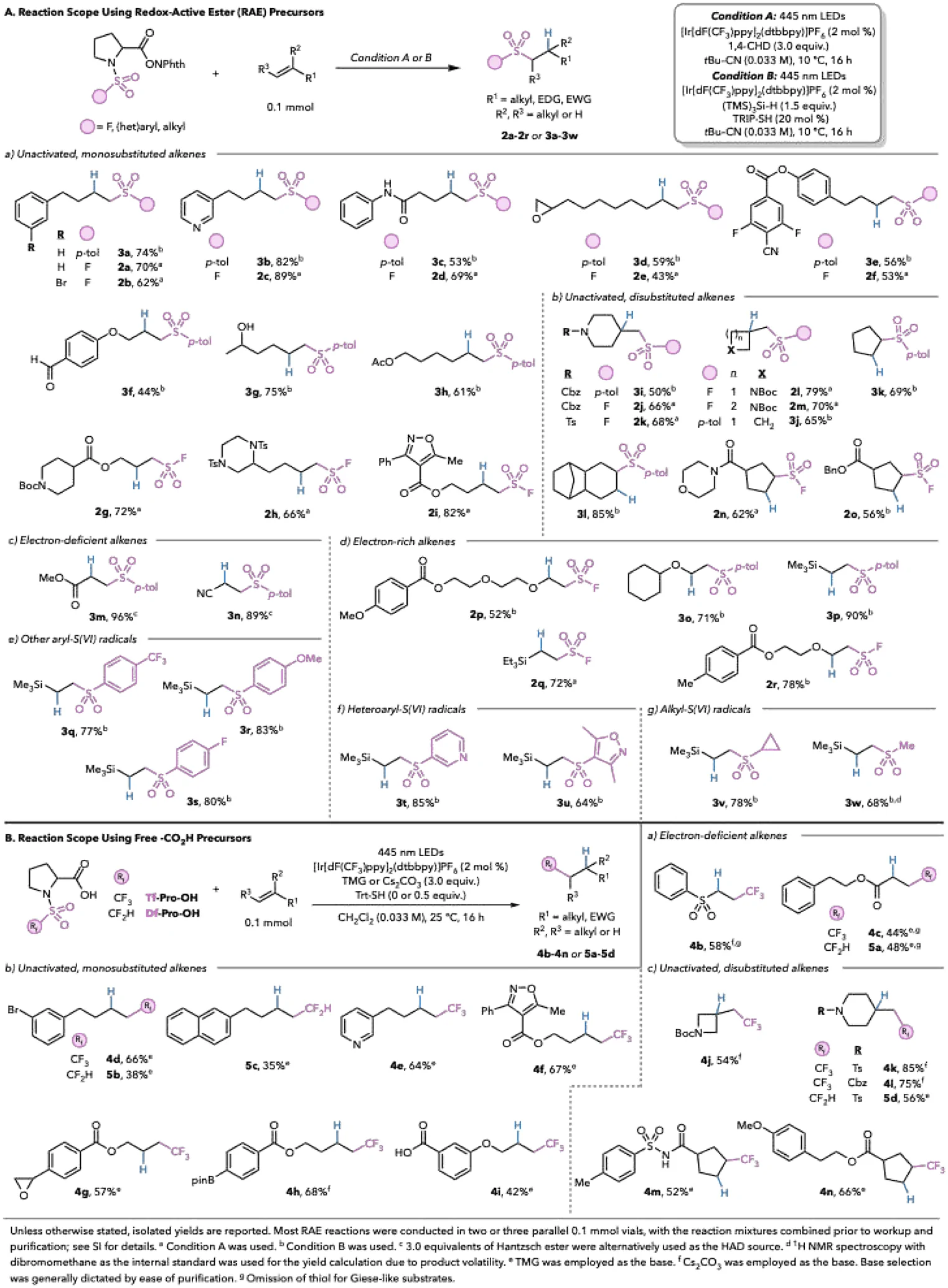
Scope of the reaction methodology.

Conditions were also optimized for trifluoromethylation of **1a** (Fig. 3B, entries 5–10). As radical-polar crossover (Fig. 6Ab, *vide infra*) is less favored for the unstabilized alkyl radical generated by addition of **•**CF_3_ to **1a**, HAT reagents were screened. Evaluation of thiol additives using TMG identified trityl thiol (Trt-SH, entry 9), a rarely employed HAT donor in photoredox catalysis, as the optimal additive, affording the desired product **4a** in 61% yield. Increasing the temperature from 10 to 25 ”C improved the yield of **4a** to 79% (entry 10) while maintaining homogeneity; these conditions were adopted for subsequent studies. Remarkably, all transformations in Fig. 4 employed a single iridium photocatalyst, despite engaging distinct oxidative and reductive activation manifolds, underscoring the convergent design of redox-release, where formation of the radical does not require catalysts with different redox potentials.

## Reaction Scope

With optimized conditions established for both oxidative and reductive activation manifolds, electron-neutral mono- and disubstituted alkenes were evaluated using **FS-Pro-RAE** and **Ts-Pro-RAE** as radical precursors (Fig. 4A, a-b). Condition A proved effective for **FS-Pro-RAE**, while higher yields were obtained with **Ts-Pro-RAE** using the slightly modified PRC Condition B. The chemistry was tolerant of groups that included pyridines (**2c**, **3b**), amides (**2d**, **3c**), epoxides (**2e**, **3d**), nitriles and esters (**2f**, **3e**, **3h**), an aldehyde (**3f**), an isoxazole (**2i**), and aryl halides (**2b**, **2f**, **3e**) in good-to-excellent yields. Protected amines bearing *tert*-butyloxycarbonyl (Boc), benzyloxy-carbonyl (Cbz), or tosyl (Ts) groups also underwent efficient hydro(fluoro)sulfonylation to afford the corresponding products (**2g**-**h**, **2j-m**, **3i**). Likewise, disubstituted alkenes delivered carbo- and heterocycles bearing -Ts and -SO_2_F handles (**2j-o**, **3i-l**).

While radicals derived from unactivated alkenes are sufficiently electrophilic to engage in polarity-matched HAT employing Condition A, α-heteroatom-substituted radicals formed from enol ethers are more nucleophilic, resulting in unfavorable HAT. Consistent with this hypothesis, the use of PRC Condition B (Fig. 6Ac, *vide infra*, Fig. 4Ad) restored efficient HAT to deliver **2p** and **2r** in good yields. To our knowledge, these are the first reported hydrofluorosulfonylations of enol ethers. Likewise, hydrosulfonylation of electron-rich and electron-poor alkenes with •Ts (Fig. 4Ac,d) furnished sulfones **3m-p** in good-to-excellent yields. Notably, these results represent rare examples of hydrosulfonylation of vinyl silanes and enol ethers.^31^

The generality of redox-release was further demonstrated by varying the transferable S(VI) group in radical additions to vinyltrimethylsilane. Aryl sulfonyl radicals beyond •Ts (Fig. 4Ae) efficiently afforded **3q**-**s**, as did heteroaryl sulfonyl radicals (Fig. 4Af). Finally, alkyl sulfonyl radicals (Fig. 4g) were readily accommodated to afford **3v** and **3w** in good yields. Together, the studies in Fig. 4A demonstrate that a common reductive activation manifold furnishes chemically distinct S(VI) radicals from a common proline-derived scaffold; while the identity of the transferable S(VI) group does not dictate the activation conditions, optimal HAT conditions are influenced by the polarity of the alkene-derived carbon-centered radical following addition.

The versatility of oxidative redox-release manifold beyond S(VI) functionalization was studied to access valuable fluoroalkanes (Fig. 4B). Hydrotrifluoromethylation with **Tf-Pro-OH** proceeded efficiently with electron-deficient Giese acceptors, including vinyl sulfone (**4b**) and acrylate (**4c**) derivatives. Unactivated terminal alkenes bearing aryl bromide (**4d**), pyridine (**4e**), isoxazole (**4f**), epoxide (**4g**), aryl boronate (**4h**), carboxylic acid (**4i**), and ester (**4f**-**h**) groups were also readily accommodated, establishing compatibility across both activated and unactivated alkenes. The scope extends to disubstituted alkenes, including four- to six-membered saturated nitrogen heterocycles protected as their Boc (**4j**), Ts (**4k**), or Cbz (**4l**) derivatives, as well as cyclic alkenes containing anisole, ester, and N-acyl sulfonamide functionality (**4m-n**). An analogous **Df**-**Pro-OH** precursor gave hydrodifluoromethylation of electron-poor (**5a**) and electron-neutral (**5b-d**) alkenes, highlighting that the platform is not limited to S(VI) radicals.

## Synthetic Utility

**Fig. 5.**
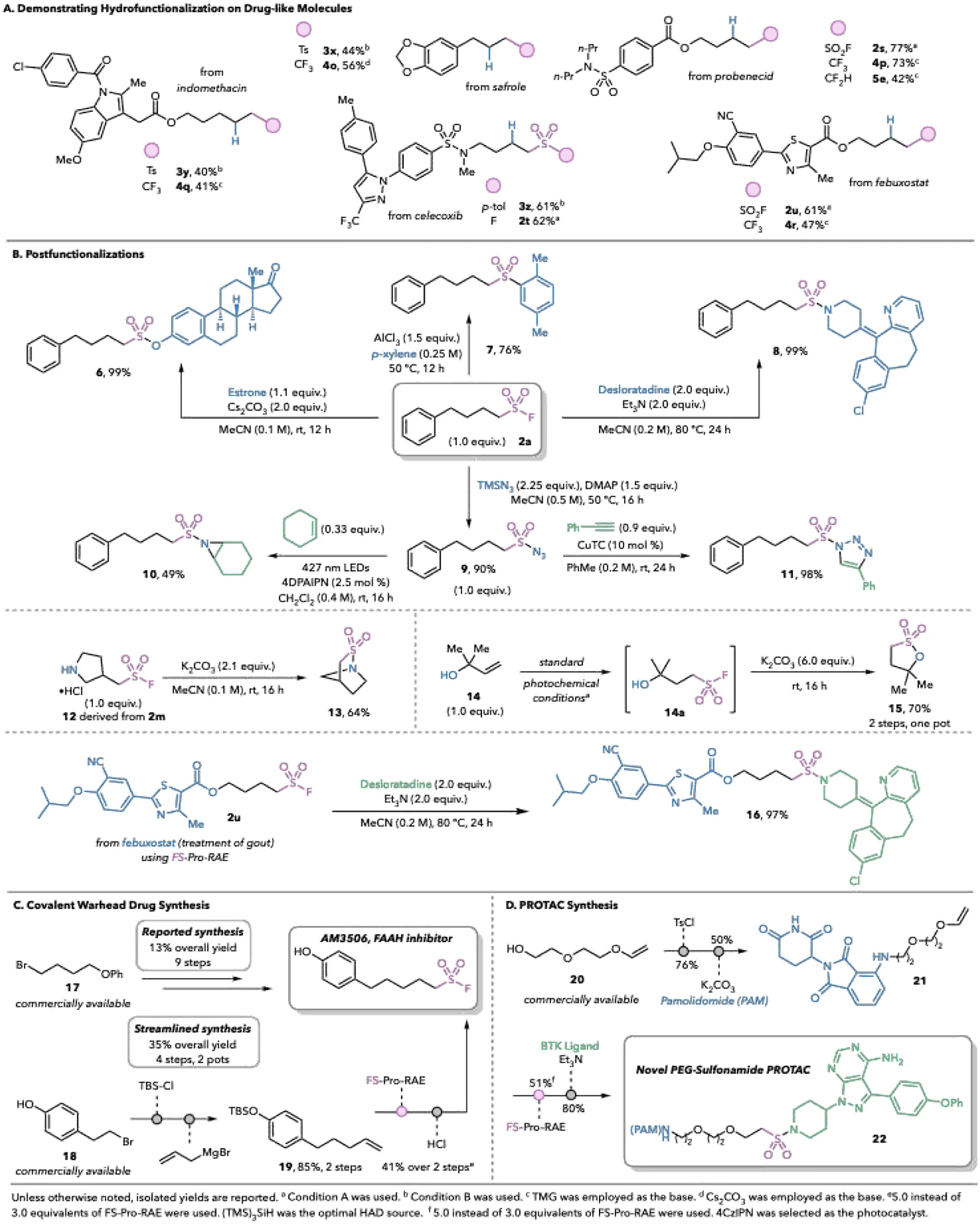
Synthetic utility of alkene hydro(fluoro)sulfonylation and hydrofluoroalkylation. (**A**) Late-stage functionalizations of drug molecules and natural products. (**B**) Post-functionalizations. (**C**) Application to the synthesis of a covalent warhead drug. (**D**) Application of hydrofluorosulfonylation to the synthesis of a novel PROTAC.

Alkenes derived from structurally complex pharmaceuticals and natural products (Fig. 5A) underwent late-stage hydrofunctionalization to install -SO_2_F, -Ts, -CF_3_ and -CF_2_H groups efficiently across diverse molecular scaffolds, underscoring the broad applicability and functional group tolerance of redox-release. Representative examples included the natural product safrole (**3x, 4o**) and the pharmaceuticals probenecid (**2s**, **4p**, **5e**), indomethacin (**3y**, **4q**), celecoxib (**2t**, **3z**), and febuxostat (**2u**, **4r**). Importantly, all four radical classes could be introduced in the presence of diverse medicinally relevant functional groups.

Alkyl sulfonyl fluorides are versatile synthetic linchpins for downstream diversifications using SuFEx chemistry (Fig. 5B). For example, reaction of **2a** with estrone and the antihistamine desloratadine provided the corresponding sulfonate (**6**) and sulfonamide (**8**) in near-quantitative yields. Notably, **2a** also participated in a rare Friedel–Crafts arylation of *p*-xylene to afford the sulfone **7**.^32^ One-step conversion of **2a** to the sulfonyl azide **9** enabled photochemical nitrene transfer to give **10** or Cu-catalyzed ‘click’ chemistry to yield **11**.^33^ Intramolecular SuFEx on **12** delivered a bridged bicyclic sultam **13**, while the compatibility of hydrofluorosulfonylation with free alcohols enabled a two-step, one-pot sequence from **14** to furnish sultone **15** in 70% overall yield. Finally, the product of late-stage hydrofluorosulfonylation of febuxostat **2u** was subjected to SuFEx coupling with desloratadine to give the drug conjugate **16** in 97% yield.

The utility of the redox-release platform for target-oriented synthesis was demonstrated in a streamlined preparation of the FAAH inhibitor AM3506 (Fig. 5C). Whereas the previously reported synthesis required nine steps and proceeded in only 13% overall yield, direct hydrofluorosulfonylation of **18** gave access to AM3506 through a two-pot, four step process in 35% overall yield.^34^ This concise route highlights how programmable installation of alkyl sulfonyl fluorides can simplify the synthesis of medicinally relevant targets. Versatility was showcased through the synthesis of proteolysis-targeting chimeras (PROTACs), bifunctional molecules that induce selective degradation of proteins by recruiting them to E3 ubiquitin ligases (Fig. 5D).^35–37^ Leveraging the unprecedented hydrofluorosulfonylation of enol ethers enabled by PRC, a PEG-linked pomalidomide (PAM) derivative **21** underwent late-stage hydrofluorosulfonylation and subsequent SuFEx coupling with an ibrutinib-derived BTK ligand to afford PROTAC **22** in 80% yield, featuring a novel PEG-sulfonamide linker. This modular strategy illustrates how the unique scope enabled by PRC unlocks previously inaccessible linker architectures, while the exceptional functional-group tolerance of redox-release creates opportunities for medicinal chemistry.

## Mechanistic investigations

**Fig. 6.**
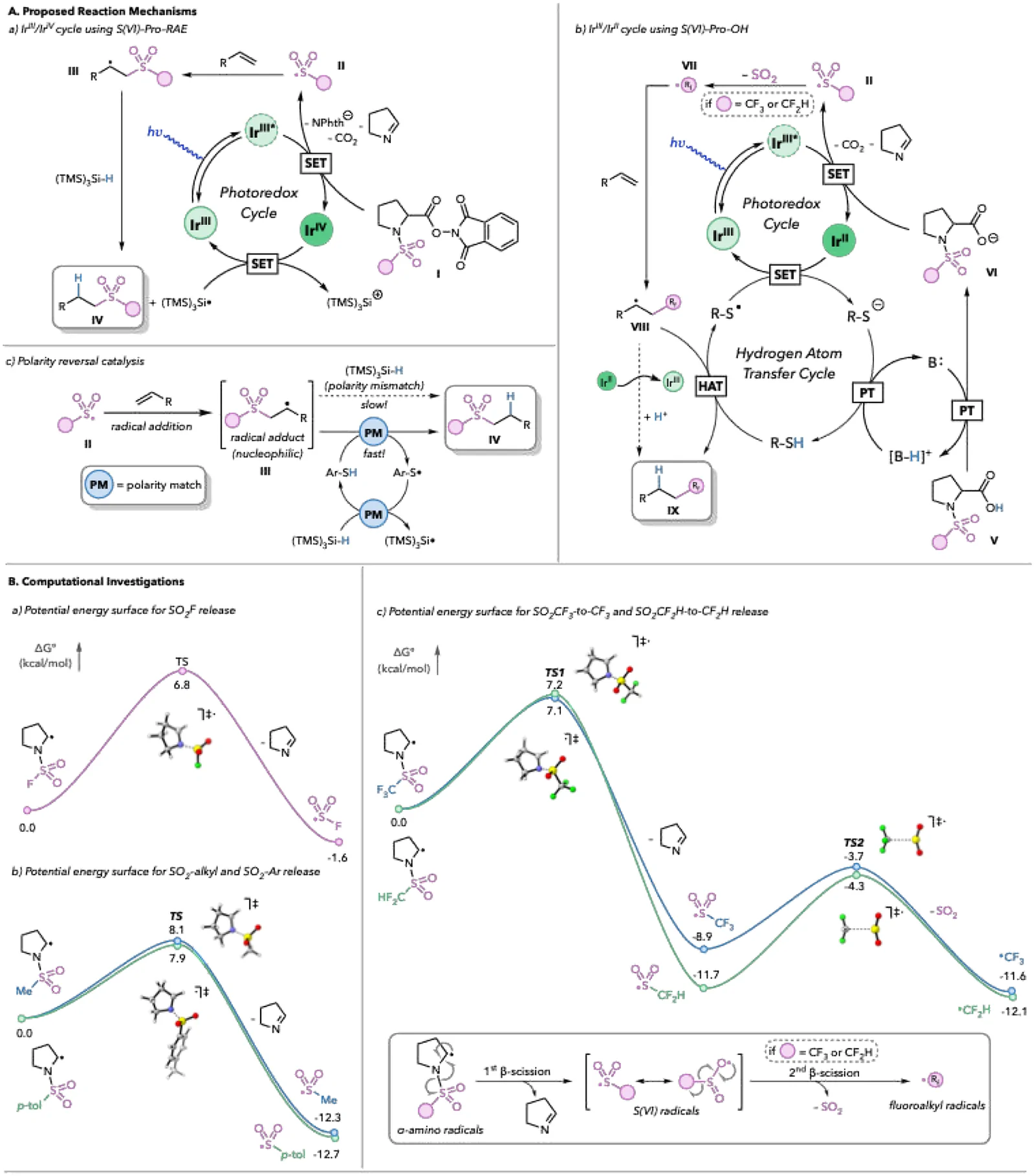
(**A**) Proposed reaction mechanisms for **a**) a) IrIII/IrIV cycle using S(VI)–Pro–RAE. **b**) IrIII/IrII cycle using S(VI)–Pro–OH. **c**) Polarity reversal catalysis. (**B**) Computed reaction energy profiles for radical fragmentation of α-amino sulfonamides. **a**) Generation of fluorosulfonyl radicals. **b**) Generation of alkyl- and aryl-sulfonyl radicals. **c**) Generation of fluoroalkyl radicals. The inset depicts the proposed β-scission pathway connecting α-amino radicals to sulfur-centered and fluoroalkyl radicals. [SMD(MeCN or DCM)-(U)M06-2X/6-311++g(2d,p)//SMD(MeCN or DCM)-(U)B3LYP/6-311+g(d,p)].

Proposed mechanisms for S(VI) radical transfer under both reductive and oxidative conditions are shown in Fig. 6Aa,b, along with the PRC cycle (Fig. 6Ac). Excitation of the Ir photocatalyst promotes single-electron reduction of RAE **I** to a radical anion, which rapidly decarboxylates to afford an α-amino radical. Subsequent β-scission releases S(VI) radical **II** and an imine. Addition of **II** to the alkene gives a carbon-centered radical **III**, which undergoes HAT to afford **IV**. In the complementary oxidative manifold (Fig. 6Ab), deprotonation of **V** and single-electron oxidation by Ir(III)* trigger decarboxylation to release the S(VI) radical **II**. Addition of **II** to the alkene and subsequent HAT affords the product. For **Tf-Pro-OH** (Fig. 2A, *vide supra*), the initially generated S(VI) radical **II** extrudes SO_2_ to furnish a fluoroalkyl radical **VII**, which adds to the alkene to give **VIII** and ultimately product **IX**. Addition of TEMPO suppressed product formation under both oxidative and reductive conditions. LC-MS analysis of the product was consistent with trapping of the α-amino radical by TEMPO (see page S25 in the Supporting Information).

While redox-release provides a unified activation manifold for radical generation and addition to an alkene, productive quenching of the resulting carbon-centered radical depends on its electronic properties. Carbon-centered radicals possessing greater nucleophilic character undergo inefficient HAT with TMS_3_SiH or 1,4-CHD due to an unfavorable polarity match. In these cases, PRC using a thiol HAT mediator promotes efficient HAT and restores reactivity (Fig. 6Ac). These observations support a common polarity-matching rationale across chemically distinct radical classes. By contrast, oxidative tri- and difluoromethylation of Giese acceptors proceed via radical polar crossover, where reduction of the stabilized carbon-centered radical regenerates the photocatalyst. The resulting carbanion is subsequently protonated to furnish **IX**.

To gain additional insight into the mechanism of the redox-release fragmentation cascade, DFT calculations were performed on the proposed β-scission pathways from the common α-amino radical intermediate.^38,39^ The energy profile for a fluorosulfonyl derivative (Fig. 6Ba) reveals that homolytic β-scission proceeds through a single transition state (TS) with an activation free energy of 6.8 kcal/mol. Formation of •SO_2_F is slightly exergonic (ΔG = −1.6 kcal/mol), indicating that cleavage of the C–S bond is both kinetically accessible and thermodynamically favorable. Addition of •SO_2_F to an unactivated alkene proceeds with an activation barrier of only 0.4 kcal/mol (see Section XII in the Supporting Information). Fragmentation of the mesyl and tosyl derivatives (Fig. 6Bb) requires slightly higher activation barriers (ΔG^‡^ = 8.1 and 7.9 kcal/mol, respectively), but are readily accessible. Formation of the sulfonyl radicals is highly exergonic (ΔG = −12.3 and −12.7 kcal/mol), rendering β-scission irreversible. These calculations support the central design principle of redox-release, where β-scission proceeds with similarly low barriers across structurally distinct S(VI) substituents. This renders radical generation largely insensitive to the identity of the transferable fragment despite substantial differences in thermodynamic stability.

Fluoroalkylsulfonyl proline derivatives undergo two consecutive β-scissions (Fig. 6Bc). Initial cleavage to generate a fluoroalkylsulfonyl radical proceeds with low activation barriers (ΔG^‡^ ≈ 7.1 kcal/mol) for both SO_2_CF_3_ and SO_2_CF_2_H-substituted prolines, providing the sulfonyl radical intermediates (ΔG = −8.9 and −11.7 kcal/mol, respectively). Subsequent extrusion of SO_2_ requires a modest activation barrier (ΔG^‡^ = 5.2 kcal/mol for CF_3_SO_2_• and 7.4 kcal/mol for CF_2_HSO_2_•, relative to the preceding intermediates). Overall, the fragmentation cascade is strongly exergonic to afford •CF_3_ and •CF_2_H with final free energies of −11.6 and −12.1 kcal/mol, respectively. These calculations support the proposed sequential fragmentation mechanism and extend redox-release beyond S(VI)-centered radicals to fluoroalkyl radicals.^18,19^ In summary, computations indicate that β-scission processes are associated with low activation barriers (<9 kcal mol^−1^), consistent with rapid fragmentation of the initially formed α-amino radicals.

## Conclusion

We have developed a redox-release platform to enable programmable access to diverse S(VI) and fluoroalkyl radicals from a common proline-derived scaffold through complementary redox activation manifolds. By localizing redox activation to the proline backbone, chemically distinct radicals are generated through a unified activation strategy independent of the transferable functional group. Mechanistic studies support a shared β-scission pathway underlying this generality, while synthetic applications demonstrate the utility of the resulting radicals in hydrofunctionalization, late-stage functionalization, diversification, and target-oriented synthesis. Beyond the specific transformations reported herein, we anticipate that redox-release will facilitate increasingly general platforms in which a unified activation strategy can be paired with a broad range of downstream bond-forming processes, expanding opportunities for reaction discovery and molecular synthesis.

## Methods

### General procedure for alkene hydrofluorosulfonation with FS-PRO-RAE radical precursor

#### Condition A.

A 4 mL borosilicate vial under ambient conditions was charged with [Ir[dF(CF_3_)ppy]_2_(dtbbpy)]PF_6_ (PC1) (2.2 mg, 2.0 μmol, 2 mol %) and FS-Pro-RAE (0.3 mmol, 3.0 equiv.). Pivalonitrile (3.0 mL, 0.033 M) was then added to the vial, followed by 1,4-CHD (0.3 mmol, 28.3 μL, 3.0 equiv.) and the desired alkene (0.1 mmol, 1.0 equiv.). The vial was charged with 5 small glass beads, capped, and placed in a Lumidox II photoreactor equipped with a 24-position, 18 mm spacing LED array. The chiller temperature was set to 10 ”C to keep the internal temperature below 25 ”C. The shaker was set to 500 rpm, and the 445 nm wavelength light at 2/5 intensity was turned on. After completion, the reaction mixture was concentrated to near dryness, diluted with CH_2_Cl_2_, and washed 3x with 10% aqueous K_2_CO_3_. The organic layers were combined, dried over MgSO_4_, filtered, and concentrated via rotary evaporation. The crude residue was purified by flash chromatography on silica gel using a hexanes/ethyl acetate mobile phase.

#### Condition B.

A 4 mL borosilicate vial under ambient conditions was charged with [Ir[dF(CF_3_)ppy]_2_(dtbbpy)]PF_6_ (PC1) (2.2 mg, 2.0 μmol, 2 mol %) and FS-Pro-RAE (0.3 mmol, 3.0 equiv.). Pivalonitrile (3.0 mL, 0.033 M) was added, followed by (TMS)_3_SiH (0.15 mmol, 46.3 μL, 1.5 equiv.), TRIP-SH (4.7 mg, 20.0 μmol, 20 mol %) and the desired alkene (0.1 mmol, 1.0 equiv.). The vial was charged with 5 small glass beads, capped, and placed in a Lumidox II photoreactor equipped with a 24-position, 18 mm spacing LED array. The chiller temperature was set to 10 ”C to keep the internal temperature below 25 ”C. The shaker was set to 500 rpm, and the 445 nm wavelength light at 2/5 intensity was turned on. Upon completion of the reaction, the reaction mixture was concentrated to near dryness, diluted with CH_2_Cl_2_, and washed 3x with 10% aqueous K_2_CO_3_. The organic layers were combined, dried over MgSO_4_, filtered, and concentrated via rotary evaporation. The crude residue was purified by flash chromatography on silica gel. For complete experimental details, including procedures and full characterization of all new compounds, see the Supplementary Information.

## Supplementary Files

This is a list of supplementary files associated with this preprint. Click to download.

- NatSynSIpart1Sept2026.docx
- NatSynSIpart2Sept2026.docx

## Data Availability

All data are available in the main text or in the Supplementary Information. Material includes general experimental procedures, characterization for all new compounds, CV studies and NMR spectra.
